# Oncological and Reproductive Outcomes after Fertility-Sparing Surgery in Patients with Advanced-Stage Serous Borderline Ovarian Tumor: A Single-Center Retrospective Study

**DOI:** 10.3390/jcm12185827

**Published:** 2023-09-07

**Authors:** Wei Cang, Chao Liang, Dan Wang, Xiaowei Xue, Dongyan Cao, Jiaxin Yang, Lingya Pan, Ming Wu, Junjun Yang, Yang Xiang

**Affiliations:** 1 Department of Obstetrics and Gynecology, Peking Union Medical College Hospital, Chinese Academy of Medical Sciences and Peking Union Medical College, National Clinical Research Center for Obstetric and Gynecologic Diseases, Beijing 100730, China; 2 Department of Pathology, Peking Union Medical College Hospital, Chinese Academy of Medical Science and Peking Union Medical College, Beijing 100730, China

**Keywords:** serous borderline ovarian tumor, advanced-stage, fertility-sparing surgery, recurrence, reproductive outcomes

## Abstract

Our study aimed to analyze the prognosis and reproductive outcomes of patients with advanced-stage serous borderline ovarian tumors (SBOTs) who underwent fertility-sparing surgery (FSS). This study included patients aged ≤ 45 years diagnosed with advanced-stage (International Federation of Gynecology and Obstetrics II and III) SBOTs who were treated with FSS. Conservative surgeries were performed in 65 patients with advanced-stage SBOT with a median age of 28 years (range, 16–44 years). Nine patients had invasive implants. The median follow-up was 81.7 months. Forty-six patients (70.8%) had a relapse (median time to first recurrence, 22.8 months). Thirteen patients subsequently developed recurrence as an invasive disease, and two died due to disease progression. After multivariate analysis, age < 30 years and incomplete cytoreduction were independent risk factors for recurrence. Invasive implants and postoperative residual tumors were significantly associated with shorter disease-free survival. Of 35 patients attempting to conceive, 12 underwent assisted reproductive technology. Additionally, 19 pregnancies, including 15 full-term births, were documented. FSS provides a good chance of reproductive success in women with advanced-stage SBOT who desire fertility preservation, but it has a high recurrence rate and risk of malignancy transformation. Patients with invasive implants should be strictly selected for FSS.

## 1. Introduction

Borderline ovarian tumors (BOTs) are common gynecological tumors, accounting for 10–15% of ovarian epithelial tumors, and commonly occur in young women in their mid-40s, with the onset ranging from 35–53 years [1,2]. Histopathologically, BOT cells proliferate actively without destructive stromal invasion. The two most prevalent BOT subtypes are serous and mucinous variants, accounting for 96% of cases. These subtypes manifest with different clinical characteristics and biological behaviors, which may contribute to distinct postoperative risk profiles [3,4]. Clinically, the characteristics of BOT are atypical proliferative tumor cells, late relapse patterns, and good prognosis [5]. Most patients with BOT (75%) are diagnosed early, and the lesions are commonly confined to the ovaries [6]. Serous BOT (SBOT), one of the most frequent BOT subtypes, could occur bilaterally, and extraovarian lesions are found in 20–40% of SBOTs [5,6]. A limited number of SBOT cases could develop into low-grade serous ovarian carcinoma (LGSOC). Recurrences frequently occur as SBOT and do not lower the survival rate. Fertility-sparing surgery (FSS) is generally accepted among patients with BOT, especially for patients within the reproductive age. Most studies showed the clinical efficacy of conservative surgery in early-stage patients, whereas only several scattered studies focused on advanced-stage patients, which partially revealed its feasibility and safety with a good prognosis [7,8]. However, data on uncertain prognostic factors and reproductive outcomes of FSS are lacking. Therefore, this retrospective study was conducted to show the oncological and reproductive outcomes of patients with advanced-stage SBOT who were treated conservatively from a single tertiary center in China.

## 2. Materials and Methods

### 2.1. Basic Characteristics

The medical records of patients with advanced-stage (II-III) SBOT who were treated with FSS at Peking Union Medical College Hospital (PUMCH) between January 1999 to December 2021 were screened. The Institutional Review Board of PUMCH approved the study. The information included demographic information, CA125 levels, surgical procedures, surgical stage, histology, residual mass, chemotherapy regimens, and relapse details. Two experienced pathologists reviewed and confirmed the original pathologic slides according to the 2014 and 2020 World Health Organization (WHO) criteria (BOT classification) and Bell’s criteria for peritoneal implants [9,10]. The tumor-staging system was based on the International Federation of Gynecology and Obstetrics 2014 criteria (FIGO 2014). The inclusion criteria included (i) age under 45 years, (ii) stage II/III SBOT confirmed by pathology, (iii) treated by FSS, (iv) with valid follow-up outcomes. The exclusion criteria were age > 45 years, non-serous histological component, and history of malignancy. FSS was defined as a procedure that preserves the uterus and at least one adnexa. Complete cytoreduction was defined as no residual tumor post-operatively. The indications and type of adjuvant chemotherapy were on the basis of residual diseases and pathological features including invasive implants and lymph node involvement. Patients were followed up by outpatient visits and telephonic interviews. We collected data, including details of recurrences and reproductive outcomes. Relapse was defined as the detection of SBOT or LGSOC at the surgery for suspected recurrence. Disease-free survival (DFS) was defined as the time from the initial surgery to recurrence or censoring, whereas overall survival (OS) was defined as the time between the date of surgery and death or censoring.

### 2.2. Statistical Analyses

A single-variable Cox proportional hazard model was used to analyze the association between prognostic factors and recurrence. Variables with *p* < 0.05 were included in the multivariate analysis. Hazard ratios were calculated for potential risk factors for relapse. Survival curves were calculated using the Kaplan–Meier method and compared with the log-rank test. All tests were two-sided, and *p* = 0.05 indicated statistical significance. SPSS software version 21.0 was used for all statistical analyses.

## 3. Results

### 3.1. Patients’ Characteristics

During the study period, 65 patients with FIGO stage II and III SBOT who underwent FSS in our hospital were selected from the overall database. The median age was 28 years, and the median follow-up was 81.7 months (range, 9.8–285.4 months). Table 1 shows the clinicopathological characteristics. Of the patients who underwent FSS, 34 and 31 were diagnosed with FIGO stage II (52.3%) and III SBOTs (47.7%), respectively. Unilateral salpingo-oophorectomy and contralateral cystectomy (USO + CC) were performed in 25 patients with BOTs, and bilateral cystectomy (BC) was performed in 31 patients. Of nine patients with unilateral disease, three and six patients underwent unilateral cystectomy (UC) and USO, respectively. Invasive implants were seen in nine (13.8%) patients. Moreover, 44 (67.7%) patients achieved complete cytoreduction. Chemotherapy was administered in 15 patients after initial surgery, and chemotherapy regimens included single-agent platinum, paclitaxel/platinum, and platinum/cyclophosphamide, with treatment duration ranging from 1–8 cycles. Table A1 shows the surgical procedures.

### 3.2. Oncological Outcomes

The total recurrence rate and median DFS were 70.8% (46/65) and 22.8 months (range, 3.0–185.7 months), respectively. Moreover, 33 (71.7%) patients relapsed with non-invasive diseases. Of 46 (78.2%) patients with recurrence, 36 underwent conservative re-operation during the first relapse. Thirteen patients (20.0%) had recurrences as invasive diseases (six with invasive implants and seven with LGSOC). Table 2 shows the characteristics of patients with invasive recurrences.

**Figure 1:**
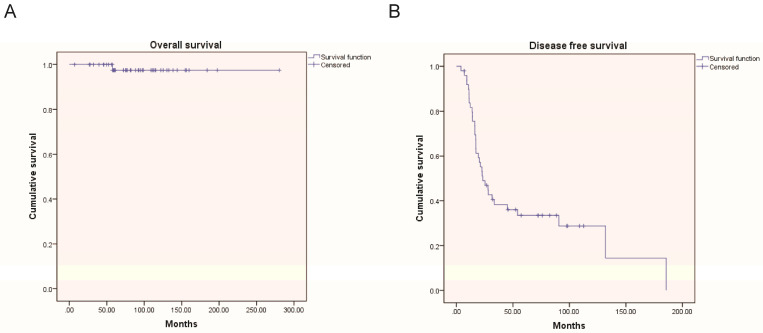
Survival curves for overall survival (**A**) and disease-free survival (**B**). OS: overall survival, DFS: disease-free survival.

We analyzed potential associations between clinicopathological factors and recurrence (Table 3). In univariate analysis, age < 30 years, FIGO III, and incomplete cytoreduction were significantly associated with higher recurrence rates (*p* = 0.011, 0.031, and 0.014, respectively). In multivariate analysis, age < 30 years and residual tumor after initial surgery were identified as independent risk factors for relapse (*p* = 0.013, 0.013, respectively). Shorter disease-free survival was observed in patients with invasive implants and residual tumors using a Cox proportional hazard model (Table 4). Except for two patients who died because of LGSOC, all patients survived. The 5-year OS and DFS rates were 97.0% and 38.5%, respectively. Figure 1 shows the Kaplan–Meier curves for DFS and OS.

### 3.3. Reproductive Outcomes

**Figure 2:**
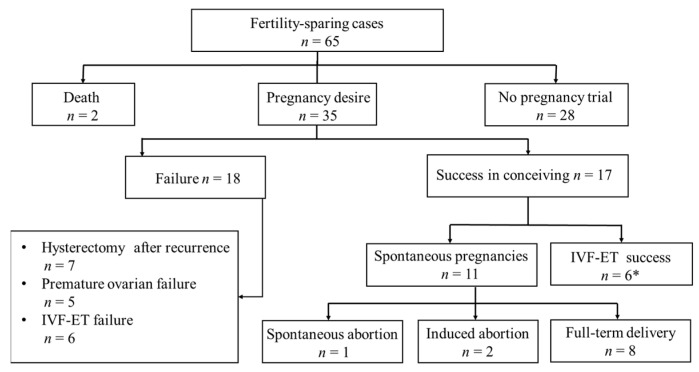
Flowchart representing reproductive outcomes. * Including five live births and one still in pregnant.

Thirty-five (53.8%) patients desired to conceive. Of these, 17 became pregnant, with 15 healthy live births in 13 women (Figure 2). Two patients had two full-term deliveries. Additionally, one woman was seven months pregnant by the end of the follow-up. One had a spontaneous abortion, and two underwent termination of pregnancy. Twelve women underwent ART, and five had at least one live-born infant. Two women underwent radical surgeries for SBOT recurrence after delivery. The median age of patients with pregnancies was similar to that of infertile patients (26 versus 27 years). The median interval between the initial operation and the first parturition was 23.7 months.

## 4. Discussion

Existing international guidelines recommended that fertility-sparing surgery is the standard approach in young patients with early-stage BOTs [10]. However, there is still no consensus on the use of FSS in cases of advanced-stage diseases. There are very few studies about conservative management, and most of those did not specifically focus on advanced-stage SBOTs [11,12,13].

Our study, conducted in a tertiary oncological referral center, is the largest single-center series investigating the oncological and reproductive outcomes of patients with advanced-stage SBOT with fertility preservation, with a median follow-up of up to 81.7 months. The 5-year OS rate of patients with advanced-stage SBOT who underwent FSS was 97.0%, which was similar to previous studies. Although most patients with advanced SBOT have a favorable prognosis, the recurrence risk should be noted. Several prognostic factors were associated with recurrence risk, including invasive implants and surgical approaches [14,15].

Invasive implants have been found in only a minority of BOT patients [16,17]. In 2014, invasive implants were considered extra-ovarian LGSOCs after WHO revised the classification of gynecological tumors [18]. In practice, European Society of Gynecological Oncology/European Society for Medical Oncology (ESGO/ESMO) and National Comprehensive Cancer Network (NCCN) do not support the terminology because they consider patients with invasive implants as different from those of classic advanced-stage LGSOCs in terms of survival of clinical management. FSS could be considered in selected SBOT patients with invasive implants according to international guidelines [10,19]. Data on conservative treatment of SBOTs with invasive implants are limited. To date, only 31 SBOT patients with invasive implants have been reported to receive FSS. In our study, nine patients with invasive implants had at least one recurrence. Moreover, six patients had a relapse with invasive diseases (two patients with LGSOCs), and one died. The median disease-free interval in patients with invasive implants is significantly shorter than in those with non-invasive implants (9.3 months versus 28.1 months, HR = 0.525, *p* < 0.001). Although the variable analysis did not consider implant type as a risk factor for recurrence due to the limited number of cases with invasive implants, data on DFS suggested the prognostic importance of invasive implants in patients undergoing FSS. Indications for FSS in patients with invasive implants should be strictly mastered. However, a large series should be conducted to explore better surgical options for patients with invasive implants.

The residual disease is an important independent prognostic factor in SBOTs with peritoneal implants [20,21]. In addition, Cytoreductive surgery is recommended in advanced-stage SBOT [10]. Complete rection of all the visible lesions is imperative for both staging and management. Our results emphasized the complete surgical excision of any lesions, which was associated with lower recurrence risk and extended DFS (Table 3 and Table 4). In our study, 14 of fifteen patients with adjuvant chemotherapy relapsed after initial surgery. The relative high recurrence rate is largely due to inherent selection bias in patients receiving adjuvant chemotherapy for more serious condition of tumors. Adjuvant chemotherapy failed to lower the relapse rate or improve the survival rate in SBOTs, even in advanced stages. There is no corroborating evidence to support adjuvant chemotherapy to date. Therefore, cytoreduction remains as the keystone for clinical benefit. To achieve complete cytoreduction, adequate preoperative evaluation should be performed to detect the location and extent of metastatic diseases for the complete removal of tumors. Teams offering cytoreductive surgery should be specialized and experienced. A careful exploration for abdominopelvic cavities, especially the diaphragm and pouch of Douglas, during operation is helpful to allow precise excision of small lesions, optimizing the chance of complete removal. At the same time, ovarian blood vessels need to be cautiously preserved for protection of ovarian function.

The present study showed that the recurrence rate significantly increased among childbearing women aged <30 years. Fotopoulou et al. [22] found that increasing age (per 10 years) seemed to have a protective effect against relapses in BOT patients. The research of Uzan et al. proposed that the young age of 30 may serve as a hallmark for predicting prognosis in stage I borderline ovarian tumors [23]. In the previous series, the role of age in advanced-stage SBOT received little attention. Our study showed no significant difference in the proportion of complete cytoreduction between the two age groups, indicating that the higher recurrence rate in young patients was not influenced by age distribution. Thus, the follow-up of young patients needs to be addressed considering their higher risk of recurrence and intention to conceive.

Many studies have assessed the effects of adnexectomy on tumor results and fertility outcomes. Fang et al. [4] concluded that USO was superior to cystectomy in reducing the relapse rate (24%). Moreover, the recurrence interval of patients undergoing USO was longer than those who received cystectomy. Vasconcelos et al. [24] reported that USO was significantly favored over cystectomy in terms of recurrence reduction. A recent phase III trial concerning BOTs demonstrated that bilateral cystectomies increased the fertility rate without increasing the recurrence rate compared with unilateral adnexectomy and a contralateral cystectomy [25]. In our study, different surgical methods for preserving fertility presented a similar relapse tendency. While the ovary volume plays a key role in fertility, the necessity of adnexectomy is debatable.

Concerning the debate of surgical approaches (laparotomy versus laparoscopy), no significant difference was found in the postoperative recurrence rates between laparoscopy and laparotomy from our study, which was consistent with earlier findings [11,15]. This may confirm the feasibility of laparoscopy. Vandenput et al. [26] pointed out that the high incidence of peritoneal recurrence was caused by less complete resection and incomplete laparoscopic visualization. However, risk factors of tumor rupture, intraperitoneal dissemination, and port-site implantation caused by laparoscopy among advanced-stage patients cannot be ignored.

With regard to pregnancy outcomes, 48.6% (17/35) of young women became pregnant after attempting to conceive following conservative treatment. Interestingly, these women who became pregnant spontaneously were either disease-free or completed childbearing before the first recurrence. The mean time between initial surgery and parturition was comparable to the disease-free interval. Therefore, the optimal timing for pregnancy might be one year after first surgery, which allows sufficient recovery post-operatively and avoid the peak time of recurrence. Considering repeated FSSs is necessary if patients have not finished childbearing before the first relapse. Besides, five women had successfully given birth to healthy babies through ART and they survived with no evidence of disease. However, whether ovarian stimulation would increase the risk of disease progression remains unclear [27,28]. Further evidence is needed for ART in advanced-stage SBOTs. Hence, an oncofertility consultation prior to fertility-sparing surgery is essential in order to assess reproductive status and prepare for postoperative pregnancies.

Our study has several limitations. First, this is a retrospective study conducted in a tertiary center. Some patients were initially diagnosed elsewhere with SBOT and then transferred to our hospital for further management. Our study’s relatively higher recurrence rate might be partly due to referral bias. Second, the limited sample size of our study may affect the generalizability of the results because of the rarity of the disease. Finally, relative shorter follow-up among several patients could result in an underestimation of recurrent risk. The follow-up time should be longer to better understand long-term outcomes, as the present evidence shows that relapse risk could still settle at a high level even after 10-year follow-up [29]. Nevertheless, the present study was the largest series of advanced-stage SBOTs undergoing fertility-preserved surgeries in Asian populations.

## 5. Conclusions

In a summary, despite the high recurrence rate, FSS could be selected in childbearing SBOT patients diagnosed at advanced stage with favorable reproductive outcomes and no impact on overall survival. Age distribution has been validated to be associated with risk of recurrences. Moreover, patients could benefit from complete cytoreductive surgery. Fertility-sparing treatment should be cautiously considered in those with invasive implants. Prospective studies with larger sample sizes are needed in the future to clarify unresolved issues with FSS for advanced-stage SBOTs.

## Figures and Tables

**Table 1:** Clinicopathologic characteristics in patients with advanced-stage SBOT.

Characteristics	*n* or Median	% or Range
Age at diagnosis (y)	28	16–42
Tumor size (cm)	7.8	2.4–30
CA125 (U/mL)	294.8	27.6–1953
Preoperative pregnancy		
Yes	27	41.5
No	39	58.5
Previous live birth		
Yes	11	16.9
No	54	83.1
Laterality		
Unilateral	9	13.8
Bilateral	56	86.2
FIGO stage		
II	34	52.3
III	31	47.7
Surgical approach		
Laparotomy	37	56.9
Laparoscopy	29	43.1
Micropapillary		
Yes	23	35.4
No	42	64.6
Invasive implants		
Yes	9	13.8
No	56	86.2
Chemotherapy after surgery		
Yes	15	23.1
No	50	76.9

**Table 2:** Characteristics of patients with invasive recurrence.

Patient	Age (yr)	FIGO Stage	Surgery	DFS (Months)	CA125 (U/mL) before Invasive Recurrence	Treatment after Recurrence	Adjuvant Treatment	Recurrence Histology	Outcome	Time To Death (Months)
1	28	IIIC	BC	10.7	69.1	RCRS	TC × 6	LGSOC	AWD	
2	23	IIIC	USO + CC	9.1	140.6	FSS→RCRS	TC × 8	Invasive implants	NED	
3	25	IIIC	USO + CC	7.1	20	FSS→RCRS	TC × 8	LGSOC	AWD	
4	22	IIIC	USO + CC	22.6	164	RCRS	None	Invasive implants	NED	
5	36	IIB	UC	9.2	91.3	FSS→RCRS	TC × 4	Invasive implants	NED	
6	28	IIIC	USO + CC	17.1	393.7	FSS→RCRS	Letrozole	LGSOC,	NED	
7	40	IIB	USO + CC	21.3	1675	RCRS	TC × 3	LGSOC,	Death	57.7
8	29	IIIB	BC	6.5	35	RCRS	None	Invasive implants	NED	
9	22	IIB	BC	3.0	303.2	RCRS	TC × 4	LGSOC	Death	81.7
10	30	IIIC	BC	6.1	216.5	FSS	None	LGSOC	AWD	
11	27	IIA	USO + CC	4.2	251	FSS→RCRS	TC × 8	Invasive implants	AWD	
12	33	IIB	UC	9.3	279.8	RCRS	None	LGSOC	NED	
13	18	IIIB	BC	16.3	14.5	FSS	None	Invasive implants	AWD	

UC: unilateral cystectomy; BC: bilateral cystectomy; USO + CC: unilateral salpingo-oophorectomy+ contralateral cystectomy; TC: Paclitaxel + platinum; DFS: disease-free survival; RS: radical surgery; FSS: fertility-sparing surgery; RCRS: repeat cytoreductive surgery; LGSC: low grade serous carcinoma; LGSOC: low grade serous ovarian carcinoma; NED: no evidence of disease; AWD: alive with disease.

**Table 3:** Risk factors for recurrence in patients with advanced-stage SBOT.

Factors	Univariate Analysis			Multivariate Analysis	
	Recurrence *n* (%)	HR (95% CI)	*p*	HR (95% CI)	*p*
Age ≥ 30 y			0.011		0.013
Yes	11 (50.0)	1		1	
No	35 (81.4)	4.375 (1.406–13.612)		5.390 (1.426–20.370)	
FIGO stage			0.031		0.254
II	20 (58.8)	1		1	
III	26 (83.9)	3.636 (1.123–11.765)		2.175 (0.572–8.266)	
Laterality			0.287		
Unilateral	5 (55.5)	1			
Bilateral	41 (71.9)	2.187 (0.517–9.245)			
Surgical approach			0.170		
Laparoscopy	18 (62.1)	1			
Open	28 (75.7)	2.139 (0.722–6.338)			
Surgical procedures			0. 609		
Cystectomy	25 (73.5)	1			
Other	21 (67.7)	0.756 (0.259–2.207)			
Lymphadenectomy			0.876		
Yes	8 (72.7)	1			
No	38 (70.4)	0.890 (0.209–3.802)			
Micropapillary			0.680		
Yes	17 (81.0)	1			
No	29 (65.9)	0.787 (0.252–2.457)			
Invasive implants			0.279		
Yes	9 (100.0)	1			
No	37 (66.1)	0.302 (0.034–2.639)			
Adjuvant chemotherapy			0.139		
Yes	14 (93.3)	1			
No	32 (64.0)	0.299 (0.060–1.480)			
Residual tumor after surgery			0.014		0.013
Yes	20 (95.2)	1		1	
No	26 (59.1)	0.072 (0.009–0.588)		0.060 (0.006–0.555)	

CI: confidential interval; HR: hazard ratio; FIGO, the International Federation of Obstetrics and Gynecology.

**Table 4:** Prognostic factors on disease-free survival in patients with advanced-stage SBOT.

Factors	Univariate Analysis			Multivariate Analysis	
	Median DFS (Months)	HR (95% CI)	*p*	HR (95% CI)	*p*
Age ≥ 30 y			0.032		0.076
Yes	58.6	1		1	
No	22.6	2.164 (1.070 4.376)		1.962 (0.932–4.127)	
FIGO stage			0.056		
II	31.6	1			
III	22.6	1.789 (0.984 3.247)			
Laterality			0.099		
Unilateral	97.8	1			
Bilateral	22.8	2.389 (0.848–6.734)			
Surgical approach			0.682		
Laparoscopy	25.4	1			
Open	23.3	1.135 (0.620–2.077)			
Surgical procedures			0. 136		
Cystectomy	17.2	1			
Other	33.5	0.634 (0.349–1.154)			
Lymphadenectomy			0.512		
Yes	22.6	1			
No	72.5	0.773 (0.358–1.669)			
Micropapillary			0.062		
Yes	17.3	1			
No	31.6	0.555 (0.299–1.029)			
Invasive implants			<0.001		0.003
Yes	9.3	5.252 (2.396–11.516)		3.764 (1.551–9.132)	
No	31.6	1		1	
Adjuvant chemotherapy			0.014		0.977
Yes	17.2	2.224 (1.174–4.214)		0.988 (0.444–2.200)	
No	31.6	1		1	
Residual tumor after surgery			<0.001		<0.001
Yes	16.2	4.709 (2.395–9.260)		3.903 (1.895–8.038)	
No	66.1	1		1	

CI: confidential interval; HR: hazard ratio; FIGO, the International Federation of Obstetrics and Gynecology.

## Data Availability

The data presented in this study are available on request from the corresponding author.

## Appendix A

**Table A1:** Surgical procedures of patients at the primary fertility-sparing surgeries.

Characteristics	
Surgical treatment	
Unilateral cystectomy	3
Bilateral cystectomy	25
Unilateral salpingo-oophorectomy	6
Unilateral salpingo-oophorectomy and contralateral cystectomy	31
Peritoneal cytology	19
Positive	15
Negative	4
Appendectomy	14
Lymphadenectomy	11
Omentectomy	31
Residual tumor	
No residual tumor	44
Residual disease	21
